# The *Colletotrichum acutatum* Species Complex as a Model System to Study Evolution and Host Specialization in Plant Pathogens

**DOI:** 10.3389/fmicb.2017.02001

**Published:** 2017-10-11

**Authors:** Riccardo Baroncelli, Pedro Talhinhas, Flora Pensec, Serenella A. Sukno, Gaetan Le Floch, Michael R. Thon

**Affiliations:** ^1^Laboratoire Universitaire de Biodiversité et Écologie Microbienne, ESIAB, Université de Brest, Plouzané, France; ^2^Linking Landscape, Environment, Agriculture and Food, Instituto Superior de Agronomia, Universidade de Lisboa, Lisbon, Portugal; ^3^Instituto Hispano-Luso de Investigaciones Agrarias, Department of Microbiology and Genetics, University of Salamanca, Salamanca, Spain

**Keywords:** *Colletotrichum acutatum*, evolution, fungal lifestyle, pathogenicity, anthracnose

## Abstract

*Colletotrichum* spp. infect a wide diversity of hosts, causing plant diseases on many economically important crops worldwide. The genus contains approximately 189 species organized into at least 11 major phylogenetic lineages, also known as species complexes. The *Colletotrichum acutatum* species complex is a diverse yet relatively closely related group of plant pathogenic fungi within this genus. Within the species complex we find a wide diversity of important traits such as host range and host preference, mode of reproduction and differences in the strategy used to infect their hosts. Research on fungal comparative genomics have attempted to find correlations in these traits and patterns of gene family evolution but such studies typically compare fungi from different genera or even different fungal Orders. The *C. acutatum* species complex contains most of this diversity within a group of relatively closely related species. This Perspective article presents a review of the current knowledge on *C. acutatum* phylogeny, biology, and pathology. It also demonstrates the suitability of *C. acutatum* for the study of gene family evolution on a fine scale to uncover evolutionary events in the genome that are associated with the evolution of phenotypic characters important for host interactions.

## Biology of the *C. acutatum* Species Complex

Many species belonging to the genus *Colletotrichum* are associated with plant diseases, commonly referred to as anthracnose. *Colletotrichum* spp. can affect a wide range of hosts and these pathogens are characterized by a global distribution. Virtually every botanic family cultivated is susceptible to one or more species of *Colletotrichum* and it is one of the most studied plant pathogenic fungi (Prusky et al., 2000; Latunde-Dada, 2001; Dean et al., 2012). Common hosts include many dicotyledonous plants such as strawberry, apple, citrus, and stone fruits, and major cereals such as maize and sorghum. Members of this genus produce crop losses up to 100% in many of these staple foods (Bailey and Jeger, 1992; Prusky et al., 2000; Talhinhas et al., 2005; Cannon et al., 2012).

*Colletotrichum* species employ a broad diversity of approaches to colonize and obtain nutrients from their hosts, ranging from biotrophs, nectrotrophs, hemibiotrophs to endophytes among others (Bailey and Jeger, 1992; Perfect et al., 1999; Rodriguez and Redman, 2008; Vargas et al., 2012; Prusky et al., 2013). Several *Colletotrichum* spp. are well-known as models for studying hemibiotrophy (Bergstrom and Nicholson, 1999; Perfect et al., 1999; Münch et al., 2008; O’Connell et al., 2012; Vargas et al., 2012). Hemibiotrophs initially infect through a brief biotrophic phase, which is associated with the production of large intracellular primary hyphae that can infect host cells without causing cell death. Later they switch to a necrotrophic phase during which narrower secondary hyphae are produced that spread throughout the host tissue causing necrotic lesions (Perfect et al., 1999). The scientific community has long studied the biomolecular processes that regulate this lifestyle, especially those related to the switch from biotrophy to necrotrophy (Dufresne et al., 2000; Thon et al., 2002; Wharton and Diéguez-Uribeondo, 2004; Peres et al., 2005; O’Connell et al., 2012).

The genus *Colletotrichum* has undergone frequent taxonomic changes in the past decades with the merging and addition of many species. Species concepts are still in a state of flux, however several major monophyletic clades, or species complexes, are now recognized (Cannon et al., 2012). A recent study provides an account of the 189 currently accepted species subdivided into 11 species complexes and 23 singleton species (Jayawardena et al., 2016).

*Colletotrichum acutatum* was identified by Simmonds () and validated by the same author in Simmonds (1968). Since then, a growing number of fungi have been assigned to *C. acutatum*. This was either based on the identification of new species or on the reclassification of other species, mostly from *C. gloeosporioides*, that show a high morphological similarity and an overlapping spectrum of hosts (Peres et al., 2005; Sreenivasaprasad and Talhinhas, 2005). In fact, while approximately 7% of the scientific literature dealing with *Colletotrichum* during a 10-year period from 1991 to 2000 addressed *C. acutatum*, this proportion raised to 18% in the following decade (based on a ISI Web of Science search on 1839 publications during 1991–2010 containing “*Colletotrichum*” in the title), depicting *C. acutatum* as a “popular” entity.

**FIGURE 1:**
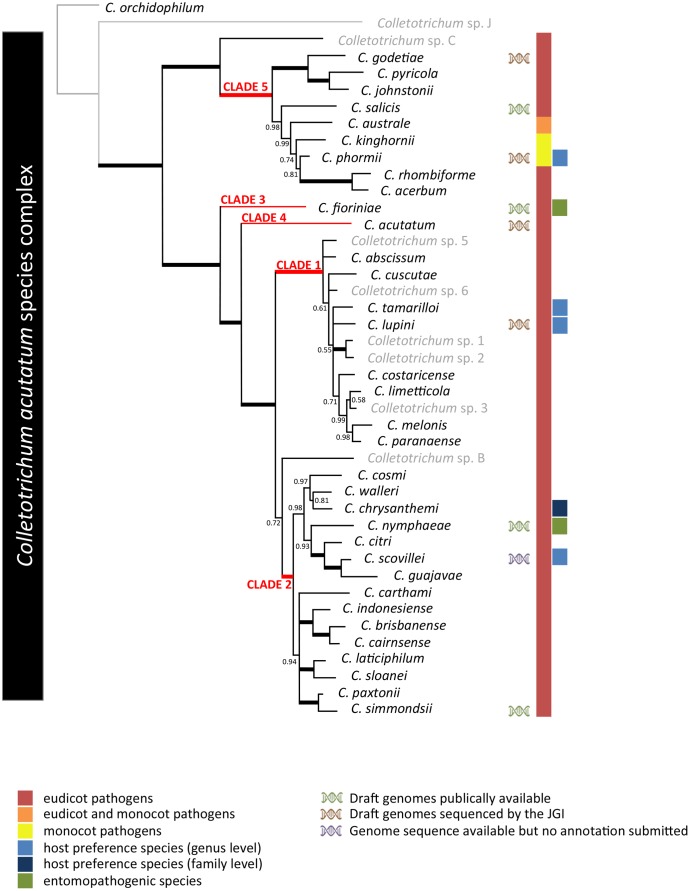
Phylogenetic analysis of the 42 *Colletotrichum acutatum* species complex strains listed in **Supplementary Table S1** based on a multilocus concatenated alignment of the ITS, GAPDH, CHS-1, HIS3, ACT, and TUB2 genes. Accepted species (Jayawardena et al., 2016) are highlighted in black while isolates not fitting with those are reported in gray. *Colletotrichum orchidophilum* was used as an outgroup.

Diversity among *C. acutatum* isolates has long been recognized (up to 4% variability in the rDNA-ITS sequences), but comparison to neighboring taxa clearly suggested this as a monophyletic group (Sreenivasaprasad et al., 1996; Damm et al., 2012; Baroncelli et al., 2016). Several intra-specific groupings were established within *C. acutatum* based on morphological, physiological, sexual, and molecular data (as revised by Sreenivasaprasad and Talhinhas, 2005), and these were compiled into eight groups (A1–A8) based on rDNA-ITS and β-tubulin 2 (TUB2) sequence analyses (Sreenivasaprasad and Talhinhas, 2005). To date 34 species are accepted (Damm et al., 2012; Huang et al., 2013; Crous et al., 2015; Bragança et al., 2016; Jayawardena et al., 2016; de Silva et al., 2017) and comprise what is now known as the *C. acutatum* species complex. Species complexes are informal designations widely used in the genus *Colletotrichum* to aggregate the huge and increasing (e.g., Pardo-de la Hoz et al., 2016) number of species described, recognizing monophyletic sub-generic groups. However, the number of species is expected to increase due to the high genetic variability of the system and to the increasing number of population studies unraveling strains that do not belong to any species described previously. The designation of infrageneric to accommodate what are now considered species complexes in the genus *Colletotrichum* would avoid confusions such as the one between *C. acutatum s.l*. and *C. acutatum* s.s. These 34 species cluster in five clades (**Figure 1**), two of which are of narrow diversity (clades 3 and 4), while the other three contain at least eight species each, with clades 2 and 5 encompassing the largest genetic diversity within the *C. acutatum* species complex.

Members of the *C. acutatum* species complex have been well-documented from agricultural and natural hosts worldwide (Peres et al., 2005; Sreenivasaprasad and Talhinhas, 2005). The species complex has a very wide host range. Meta-analyses have revealed that *C. acutatum s.l.* strains have been associated with infection on 100s of species from more than 90 genera of plants (Magnoliophyta: monocotyledons and dicotyledons, Pinophyta and Pteridophyta), along with at least two insect species (Marcelino et al., 2008; Mascarin et al., 2016) and in a few cases also with marine ecosystems (Manire et al., 2002; Namikoshi et al., 2002). There appears to be little to no evidence of co-evolution of host and pathogen. This is especially true in cultivated fruit systems such as strawberry and olive that are susceptible to many members of the species complex (Baroncelli et al., 2015b; Talhinhas et al., 2015). Although the *C. acutatum* species complex is regarded as polyphagous, the lupin anthracnose pathogen has been shown to form a well-defined, homogeneous and host-specific group, unlike other heterogeneous and polyphagous groups (Talhinhas et al., 2016). A similar situation applies to safflower (*Carthamus tinctorius*), as only strains belonging to *C. chrysanthemi* were pathogenic to safflower. Strains genetically very close but belonging to different species were not pathogenic on the same host (Baroncelli et al., 2015a). Other members of the species complex that show a strong relationship with a specific host include *C. phormii* pathogenic to *Phormium* spp. a monocot native to New Zealand and *C. tamarilloi* the causal agent of tamarillo (*Solanum betaceum*) anthracnose (Baroncelli, 2012; Damm et al., 2012).

The lifestyles employed by members of the *C. acutatum* species complex depend on many factors, including the host, host tissue infected, and the environment (Redman et al., 2001; Dieguez-Uribeondo et al., 2005; Peres et al., 2005). For example, on apples (*Malus domestica*) members of the *C. acutatum* species complex cause necrotic lesions of fruits but does not cause any symptoms on leaves (Peres et al., 2005). On sweet orange, the pathogen acts as a necrotroph on flowers and as a biotroph on leaves (Peres et al., 2005). On olive, at least six *Colletotrichum* spp. are associated with anthracnose, most frequently *C. nymphaeae*, *C. godetiae*, and *C. acutatum* (Talhinhas et al., 2005, 2009). Upon penetration of mature fruits, these species have a short biotrophic phase that is represented by multi-lobed primary hyphae, followed by an extended necrotrophic stage, leading to disease symptoms. On olive leaves and branches, the fungus is most frequently asymptomatic but capable of epiphytic growth and sporulation (Talhinhas et al., 2011). *Colletotrichum nymphaeae* is also an entomopathogen infecting the citrus orthezia scale insect, *Praelongorthezia praelonga* (Mascarin et al., 2016) while *C. fioriniae* is pathogenic toward the insect *Fiorinia externa* (Marcelino et al., 2008).

On some fruits, such as apple and blueberry (*Vaccinium* spp.), members of the *C. acutatum* species complex cause both pre-harvest and post-harvest diseases (Milholland, 1995; Jones et al., 1996; Shi et al., 1996). On these plants, asymptomatic, quiescent infections occur on unripe fruit and begin to develop necrotic lesions as the fruit ripens (Alkan et al., 2015). There are many other examples of post-harvest decays on fruits such as peaches (*Prunus persica*), almonds (*Prunus dulcis*), avocado (*Persea americana*), mango (*Mangifera indica*), papaya (*Carica papaya*), and guava (*Psidium guajava*) that develop from quiescent infections that began in the field (Agostini et al., 1992; Freeman and Shabi, 1996; Smith, 1998; Afanador-Kafuri et al., 2003).

Sexual reproduction has been identified in some *Colletotrichum* species but for most of them only the anamorph is known (Carvajal and Edgerton, 1944; Politis, 1975; Guerber and Correll, 2001). *In vitro* analyses have demonstrated that some *C. acutatum* species are heterothallic, requiring two strains of opposite mating types to complete the sexual cycle (Guerber and Correll, 2001) while others are homothallic, capable of sexual reproduction without a second strain (Talgø et al., 2007; LoBuglio and Pfister, 2008; Baroncelli, 2012; Damm et al., 2012). Phylogenetic analyses also suggest a strong relationship between monophyletic lineages and mating behavior (Baroncelli, 2012). In this case, a hypothetical homothallic ancestor and two lineage-specific events: the first being the acquisition of heterothallic capability in specific lineages and the second being the complete loss of mating behavior in other lineages. Thus, *C. acutatum s.l.* is a suitable system for studying the genetic bases of fungal mating systems and the effect of sexual behavior in genome architecture, ecological adaptation, and host association patterns.

*Colletotrichum acutatum s.l.* also shows a worldwide geographic distribution. Strains belonging to these taxa are present in diverse climatic zones worldwide (Sreenivasaprasad and Talhinhas, 2005; Damm et al., 2012). Even if different geographic areas do show particular trends in population distribution any strong connection between genetic groups or populations and their distribution has not been shown yet. However, results from previous studies do suggest Oceania as the possible origin of the complex and particularly of clade 5 (Baroncelli, 2012). This region showed the highest level of variability and strains closely related to a hypothetical ancestral population are mainly distributed in Australia and New Zealand. The low genetic variability of clade 1 and the presence of all the strains that cannot be assigned to designed species might reflect an on going speciation process. Clade 1 probably originated in South America as this geographic origin shows the highest diversity within the clade and more genetic groups and species are recently being described from this area. Interestingly only *C. lupini* as member of Clade 1 spread across the globe (over the last few decades) while all others report are mainly confined in South America. It is tempting to speculate on a cause-effect relationship between the capacity of a specific population to infect lupins and its worldwide spread, especially considering that *C. lupini* is a seed-born pathogen (Talhinhas et al., 2016). Clade 4 is represented solely by *C. acutatum sensu stricto* [i.e., the fungus identified by Simmonds (1965) in Australia as the species holotype]. The populations clustering in this taxon show limited diversity, but they can nevertheless be found on a relatively large number of hosts predominantly in Oceania and Africa (Sreenivasaprasad and Talhinhas, 2005), although recently this fungus has emerged as a pathogen associated with olive anthracnose, along with *C. nymphaeae* and *C. godetiae*, in several Mediterranean countries (Talhinhas et al., 2009; Mosca et al., 2014; Chattaoui et al., 2016), suggesting shifts in pathogen populations at global scale.

The general evolutionary trend emerging in the *C. acutatum* species complex appears to be that of distinct populations undergoing clear changes in their host-association pattern. This also might suggest a role of switching in mating behavior and changes in host association patterns. The occurrence of heterothallism seems to have influenced host range diversity. In contrast, isolates capable of homothallism (*C. salicis* and *C. phormii*) have a narrow range of hosts. The evolutionary trend suggests that the capacity of populations to exchange genetic information lead to rearrangement at the genomic level increasing genome plasticity and the host spectrum of this pathogen.

## *Colletotrichum* Genomics

Genome sequences for 28 species of *Colletotrichum* have been published in recent years, and comparative genomic studies have focused on genes associated with pathogenicity (Gan et al., 2013), host specialization (Baroncelli et al., 2016; Gan et al., 2016), transition in lifestyle between biotrophy and necrotrophy (O’Connell et al., 2012) and more recently between endophytic and parasitic lifestyles (Hacquard et al., 2016; Hiruma et al., 2016). Comparative analyses of secretomes have revealed that the majority of predicted secreted proteins have enzymatic activity, many of which are candidate effectors (O’Connell et al., 2012; Gan et al., 2013; Baroncelli et al., 2016; Sanz-Martín et al., 2016). The host range of *Colletotrichum* spp. appears to be associated with genes loss or gain in families such as those encoding carbohydrate-active enzymes (CAZymes) and proteases (Baroncelli et al., 2016). In particular, gene families encoding proteases and carbohydrate-degrading enzymes are highly expanded in *Colletotrichum* compared to other Sordariomycetes (Gan et al., 2013; Baroncelli et al., 2016), suggesting in increased importance of these gene families in *Colletotrichum* spp. infection processes. Transcriptional profiling experiments have also revealed that CAZymes, along with other classes of secreted proteins are highly modulated during the infection process, further implicating them as important players in pathogenicity (Kleemann et al., 2012; O’Connell et al., 2012).

Another important feature that has emerged from comparative studies is that lifestyle and host preference is not correlated with phylogenetic relationships (Baroncelli et al., 2016; Gan et al., 2016). A comparison of the genomes of the endophyte *C. tofieldiae* and the closely related pathogen *C. incanum* revealed that the transition to an endophytic lifestyle was associated with a reduction in the number of predicted effectors and an expansion in chitin-binding and secondary metabolism related proteins (Hacquard et al., 2016). In contrast, relatively distantly related members of the *C. acutatum* and *C. gloeosporioides* species complexes have strikingly similar repertoires of carbohydrate-active enzymes and secreted proteases (Baroncelli et al., 2016). This observation suggests that these gene families evolved recently and independently in these two phylogenetically separated lineages (Baroncelli et al., 2016; Gan et al., 2016). Further comparative analyses of the *C. acutatum* species complex genomes revealed that necrosis and ethylene-inducing peptide 1-like proteins were twice as abundant as the other fungi studied. The large number of these genes associated with leaf necrosis and immunity associated responses in Dicotyledonous plants would make the *C. acutatum* species complex a model system to study their evolution and biological role (Baroncelli et al., 2016).

## The *C. acutatum* Species Complex as a Model to Study the Evolution of Plant Pathogens

Recently, the genomes of more than 20 *Colletotrichum* species have been sequenced (O’Connell et al., 2012; Alkan et al., 2013; Gan et al., 2013, 2016, 2017; Baroncelli et al., 2014a,b, 2016; Hacquard et al., 2016; Han et al., 2016; Hiruma et al., 2016; Queiroz et al., 2017), revealing a tremendous diversity in genome architecture and gene content. While most of the studies focused on comparisons of distantly related lineages they also demonstrate the need for higher resolution taxonomic sampling in order to better understand the evolution of fungal genomes and the possible association with phenotypic characters such as host range, pathogenic lifestyle and reproductive strategy. In this context, the *C. acutatum* species complex provides a good model system, offering a variety of evolutionary closely related lineages with different phenotypic characteristics. The switch between mono and dicotyledonous hosts occurred in clade 5 (**Figure 1**) gives an example. Comparative genomics and transcriptomics of late diverging species adapted to different hosts could help us to gain an understanding of the genomic bases involved in this host switch. Another example is provided by the host specialization of certain lineages to a specific host such as *C. lupini*. The biological diversity of the *C. acutatum* species complex and the presence of very closely related species with different characteristics such as host range and spectrum and mating behavior makes *C. acutatum* a suitable model to investigate genomic signatures associated with changes in important phenotypic characters of fungal plant pathogens.

## Materials and Methods

Sequences for the genes used for phylogenetic analyses (ITS, GAPDH, CHS-1, HIS3, ACT, TUB2) were retrieved from public databases (**Supplementary Table S1**). Multiple sequence alignments were performed with MAFFT v. 7.304 (Katoh and Standley, 2013) were exported to MEGA7 (Kumar et al., 2016) where best-fit substitution models were calculated for each separate sequence dataset. The multilocus concatenated alignment was performed with Geneious 10.2.2^1^ (Kearse et al., 2012). A Markov Chain Monte Carlo algorithm was used to generate phylogenetic trees with Bayesian probabilities using MrBayes 3.2.1 (Ronquist and Huelsenbeck, 2003) for the combined sequence alignment. Models of nucleotide substitution for each gene determined by MEGA7 were used for each locus. The analysis in MrBayes ran for 5^∗^10^6^ of generations to reach a *P*-value lower than 0.01 with two parallel searches using three heated and one cold Markov chain sampled every 100 generations and 25% of the generations were discarded as burn-in.

## Author Contributions

All authors listed have made substantial, direct and intellectual contribution to the work, and approved it for publication. RB and PT drafted the manuscript; FP, SS, GLF and MT critically revised and improved the manuscript.

## Conflict of Interest Statement

The authors declare that the research was conducted in the absence of any commercial or financial relationships that could be construed as a potential conflict of interest.

## Footnotes

1 http://www.geneious.com

## Supplementary Material

TABLE S1 List of *Colletotrichum acutatum* reference strains used for the phylogeny shown in **Figure 1**, indicating species designation, strain ID, GenBank accession numbers of the sequences retrieved and used for phylogenetic analyses and references.
